# Psychosocial, neurocognitive, and physical development in Eastern European adopted adolescents with and without fetal alcohol spectrum disorder

**DOI:** 10.1111/acer.70068

**Published:** 2025-04-27

**Authors:** Pablo Carrera, Laurie C. Miller, Jesús Palacios, Maite Román

**Affiliations:** ^1^ Department of Developmental and Educational Psychology Universidad de Sevilla Seville Spain; ^2^ Faculty of Health Sciences Universidad Isabel I Burgos Spain; ^3^ Department of Pediatrics, Eliot‐Pearson Department of Child Study & Human Development, Friedman School of Nutrition Science & Policy Tufts University Boston Massachusetts USA; ^4^ GHU Paris Psychiatrie & Neurosciences Paris France

**Keywords:** fetal alcohol spectrum disorder, international adoption, neurobehavioral impairment, neurocognitive development, psychosocial adjustment

## Abstract

**Background:**

Most internationally adopted individuals have been exposed to an array of developmental risk factors, including early institutionalization and other adverse experiences. Adopted children from Eastern Europe tend to show worse neurodevelopmental outcomes than adopted individuals from other areas of origin. Previous studies have shown a high incidence of prenatal alcohol exposure and fetal alcohol spectrum disorders (FASD) among this population, a factor that may explain some of their complex needs. However, direct comparisons of adoptees (from Eastern Europe or elsewhere) with and without FASD have not been previously reported.

**Method:**

In this study, we compared 69 internationally adopted adolescents with and without FASD and 30 community adolescents in their degree of physical growth, neurobehavioral impairment, psychosocial, and neurocognitive function using standardized assessments including both parent‐reported questionnaires and performance tests. The presence of FASD or prenatal alcohol exposure was collected via parent reports.

**Results:**

Fourteen adopted adolescents (20.3% of the adopted sample) had FASD, whereas 55 adopted adolescents did not. Adopted adolescents in the FASD group showed more difficulties in several domains of psychosocial adjustment and cognitive development and lower head circumferences, compared with adopted adolescents without FASD and community adolescents, whereas in a few other areas, the pattern was less clear‐cut. Adopted adolescents without FASD also presented difficulties, although to a lesser degree.

**Conclusions:**

Adopted adolescents with FASD showed a complex profile of deficits in social communication, certain neurocognitive areas (particularly in working memory and language), and self‐regulation. However, given that internationally adopted individuals have been exposed to an array of developmental risk factors for neurodevelopment, caution is needed before assuming that the difficulties observed in adoptees from Eastern Europe derive exclusively from prenatal alcohol exposure.

## Graphical abstract

Adopted adolescents with reported fetal alcohol spectrum disorder (FASD) showed significant impairments in psychosocial adjustment, neurocognitive function, and head circumference compared to adopted adolescents without FASD and community peers. Language, working memory, and affect regulation were particularly affected. However, adopted adolescents without FASD also presented difficulties. The study underscores the need for cautious diagnosis and tailored support for internationally adopted individuals and their families, as early adversity and prenatal alcohol exposure both contribute to significant developmental challenges.

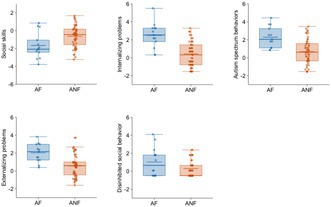

## INTRODUCTION

International adoption of children from institutions has been a common practice for decades, peaking in the first decade of the 21st century (Selman, 2024). Although their physical, cognitive, and socioemotional catchup after adoption is remarkable, children adopted from institutions often present various long‐term negative consequences of institutionalization and exposure to other early adversities (Brodzinsky & Palacios, 2023). Institutions are typically characterized by high caregiver‐to‐child ratios, rotating staff, rigid routines, and a low degree of one‐to‐one interactions (The St. Petersburg‐USA Orphanage Research Team, 2005; van IJzendoorn et al., 2020). Institutionalization has been called structural neglect, since it deprives young children of expectable experiences during sensitive periods for brain, socioemotional, and cognitive development, in particular of one‐to‐one interactions with caregivers and of the opportunity to form selective attachments due to the high staff turnover (Brodzinsky & Palacios, 2023; Nelson et al., 2023; van IJzendoorn et al., 2020). The degree of institutional deprivation varies from grossly depriving institutions in which even physical needs are not met to institutions that may meet physical needs but are psychosocially depriving (Gunnar et al., 2000).

Prior to being institutionalized, children may have also been subject to other prenatal, perinatal, and postnatal risk factors for neurodevelopment, such as undernutrition including micronutrient deficiencies (especially iron) and poor care. Some internationally adopted children have experienced physical or other forms of maltreatment prior to adoptive placement (Brodzinsky & Palacios, 2023; Johnson & Gunnar, 2011). The consequences of this accumulation of early adversities on neurodevelopment can be profound. When placed in an adoptive or foster care family, postinstitutionalized children often present severe delays in physical, cognitive, and brain development, as well as disturbances in socioemotional development, such as disinhibited social engagement or disorganized and insecure attachment (Nelson et al., 2023; van IJzendoorn et al., 2020). Placement in a family through intercountry adoption typically leads to a catchup in most developmental areas, although in some like executive functions, disinhibited social engagement, or attention‐deficit/hyperactivity disorder (ADHD), the consequences can be persistent, particularly if placement took place significantly after the infancy years (Brodzinsky & Palacios, 2023; Nelson et al., 2023; van IJzendoorn et al., 2020). This has been interpreted as an indication of sensitive periods for some areas of cognitive and socioemotional development (Nelson & Gabard‐Durnam, 2020).

Besides the timing of placement, the zone of origin may also have some relevance to the extension and degree of the developmental consequences of early adversity among internationally adopted individuals (Brodzinsky & Palacios, 2023). Postinstitutionalized adopted individuals from Eastern Europe typically show a more complex profile than adopted individuals from other regions (Miller et al., 2022). This region, especially Romania in the 90s and the Russian Federation in the early 2000s, was one of the main regions of origin for international adoptions during the peak of this practice (Selman, 2024). An element that may be particularly relevant for understanding outcomes among this specific group is prenatal exposure to alcohol and related fetal alcohol spectrum disorders (FASD; Colom et al., 2021).

The case for a high prevalence of FASD among children internationally adopted from Eastern European institutions is supported by the high proportion of pregnant mothers who drink alcohol in this region; Russia and Belarus are among the five countries with the highest prevalence of alcohol use during pregnancy (36.5% and 46.6%, respectively), while the worldwide global prevalence is 9.8% (Popova et al., 2017). The few studies analyzing the prevalence of FASD among internationally adopted individuals from Eastern Europe through diagnostic tools and multidisciplinary teams (including pediatric and neuropsychological assessments) have shown high rates of FASD. A survey of several Russian institutions analyzing only physical phenotypic features showed that 15% of the children had facial features highly compatible with full FAS and 45% had intermediate facial phenotype scores (*N* = 234; Miller et al., 2007). A Swedish study including ophthalmologic, pediatric, and neuropsychological assessments with 71 children adopted from Eastern Europe showed that 52% of the children showed FASD (Landgren et al., 2010). In Spain, one of the countries that received more adopted children from Eastern Europe (Selman, 2024), a recent study in the region of Catalonia evaluated 162 adolescents adopted from Russia and Ukraine in infancy and early childhood for FASD, including assessment of facial dysmorphia and neurobehavioral domains. The results showed that 50% of the adopted sample had features consistent with FASD (Colom et al., 2021). An additional study assessing FASD in children adopted internationally from Poland (*N* = 121) found that 31% of the children received a diagnosis within the spectrum of FASD, and adoptive parents of an additional 21% of the total sample suspected a diagnosis within the spectrum of FASD (Knuiman et al., 2015).

FASD is an umbrella including a variety of mental and physical conditions in children who have been exposed to maternal alcohol consumption during pregnancy (Lange et al., 2019; Mattson et al., 2019; Wozniak et al., 2019), associated with secondary conditions such as disrupted schooling, criminal offenses, or risky sexual behaviors (McLachlan et al., 2020). The most widely known condition within this spectrum is fetal alcohol syndrome (FAS), characterized by growth deficiencies, damage to the central nervous system, and specific facial dysmorphia (Popova et al., 2023). However, the broad and persistent consequences of prenatal alcohol exposure on neurobehavioral development can also be present in the absence of physical dysmorphia, as in the FASD alcohol‐related neurodevelopmental disorder (Cook et al., 2016; Wozniak et al., 2019). Conditions within the fetal alcohol spectrum include difficulties in neurocognitive domains (particularly in intelligence, executive functions, attention, language, learning, and memory) and behavioral or psychosocial domains (among others, in mood or affect regulation, behavior problems, adaptive functioning or social skills, and academic performance; Kully‐Martens et al., 2012; Kingdon et al., 2016; Mattson et al., 2019; Popova et al., 2023; Wozniak et al., 2019). Impairments in at least some of these neurobehavioral domains are a necessary condition for identifying FASD according to diagnostic guidelines (Cook et al., 2016; Hoyme et al., 2016). Furthermore, FASD is highly comorbid with other medical and psychological conditions, the most common being electrophysiological abnormalities in the peripheral nervous system, language disorders, chronic serous otitis, and conduct disorders, according to a metanalysis (Popova et al., 2016).

Previous research has shown that exposure to additional postnatal adversity among children with FASD predicts worse psychosocial and cognitive outcomes (Kambeitz et al., 2019; Rockhold et al., 2023). Therefore, the consequences of prenatal exposure to alcohol may compound with the effects of postnatal adversity, with a substantial overlap between some of the core behavioral manifestations of FASD and the neurodevelopmental consequences of early adversity (Rockhold et al., 2023).

Individuals with FASD and their relatives may face significant barriers to access appropriate services and support, stigma, and other issues that may cause stress and be difficult to manage (Mohamed et al., 2020). Although families caring for a son or daughter with FASD face a significant burden, most parents also recognize positive influences from them (Kautz‐Turnbull et al., 2022).

Early identification and diagnosis of FASD are important to support families and children and prevent the secondary conditions common in individuals with FASD (Bashista, 2022; Streissguth et al., 2004). However, the diagnosis of individuals without definitive facial dysmorphia is complicated by the nonspecificity of the broad neurodevelopmental impairments present in FASD. Even if children and adolescents with FASD can be accurately distinguished from typically developing children based on their neurobehavioral profiles, they cannot always be differentiated from individuals with other neurodevelopmental disorders. Thus, it has been questioned whether there is a unique neurobehavioral profile associated with FASD (Lange et al., 2019).

Additional challenges for diagnosing FASD are that the sentinel physical features associated with FASD tend to become less detectable after childhood (Jacobson et al., 2021), and, crucially, that often there are no reliable records of prenatal alcohol exposure (Brown et al., 2019). The lack of reliable records, particularly for internationally adopted individuals, poses difficulties to FASD diagnosis using classic criteria, which require documented prenatal alcohol exposure (Brown et al., 2019). In the case of individuals who have suffered significant postnatal adversity in the form of institutionalization and others, it is even more difficult, if not impossible, to discern if neurocognitive, self‐regulation, and adaptive functioning challenges are manifestations of FASD or consequences of postnatal adversity, given the overlap in the areas affected by both (Mattson et al., 2019; Nelson et al., 2023; Rockhold et al., 2023).

### The present study

In this study, we contribute to the sparse literature on the profile and special needs of adolescents adopted from Eastern Europe with FASD, as reported by their adoptive parents, by comparing them with adopted adolescents without FASD, but with comparable postnatal adversity, and a group of nonadopted adolescents from the same communities. Specifically, we examined differences among these groups in a number of neurodevelopmental domains commonly impaired in FASD, including motor skills, neuroanatomy, cognition, language, attention, executive function, memory, affect regulation, and social skills (Cook et al., 2016; Hoyme et al., 2016). Our second aim was to compare the adopted adolescents with reported FASD, the adopted adolescents without FASD but with a similar postnatal trajectory, and the community adolescents in a battery of psychosocial/behavioral, neurocognitive, and physical growth domains. We also compared the number and type of comorbid conditions and service use between the three subgroups of adolescents.

We hypothesized that adopted adolescents with reported FASD would show impairments in a greater number of neurodevelopmental domains than adopted adolescents without FASD, although we expected that this latter group would show a higher number of impairments than the community comparison group due to their exposure to other preadoption adversities. We expected that adopted adolescents with reported FASD would also have more comorbid conditions and higher service use than adopted adolescents without FASD. Broadly, we hypothesized worse outcomes in psychosocial adjustment, neurocognitive, and physical development for the adopted group with FASD as compared with the other two groups and relatively worse outcomes for the adopted group without FASD but with postnatal adversity as compared with the community group.

## MATERIALS AND METHODS

### Participants

The sample included 99 Spanish adolescents between 13 and 18 years old, including 69 intercountry adoptees (mean age = 15.82 years, SD = 1.35) and 30 community adolescents (mean age = 16.11 years, SD = 1.12) who were part of the Longitudinal Adoption and Institutionalization Study at the Universidad de Sevilla (LAIS.US). The country of origin of all the adopted adolescents was the Russian Federation. Age at adoption ranged from 9 to 61.50 months (*M* = 32.91, SD = 14.96). Most adopted adolescents resided in institutions before being adopted (59, 85.5%), for an average of 33.14 months (SD = 15.12). At the time of assessment, they had lived a mean of 13.06 years (SD = 1.25) with their adoptive families. Children were adopted between 2001 and 2006, that is, before adoptions from Russia involved mainly children with special needs (Selman, 2012).

Table 1 shows descriptive sociodemographic data for both the adopted (*n* = 69) and the community groups (*n* = 30). The adopted group had a higher proportion of males, as is usual with adoptions to Spain from the Russian Federation (AIPAME, 2013).

**TABLE 1:** Descriptive demographic information for the internationally adopted and the community group.

	Adopted	Community
	*n* (%)	*n* (%)
Adolescents demographics		
Female gender	15 (37.9)	17 (56.7)
Male gender	41 (62.1)	13 (43.3)
Parents^a^ demographics		
Female gender	60 (86.96)	30 (100)
Male gender	9 (13.04)	0 (0)
Higher education level	52 (75.36)	16 (53.3)
Family structure		
Bi‐parental	51 (73.91)	26 (86.7)
Single mother/father	18 (26.09)	4 (13.3)

^a^ Demographics are provided for the parent who completed the questionnaire.

### Procedure

Adoptive families were recruited through collaboration with international adoption agencies and regional administrations in Spain. Some adoptive families (*n* = 31) and the community group (*n* = 30) were part of the longitudinal LAIS.US study (see the official webpage for more details) and had participated in previous data collections. These families were contacted by telephone for this data collection. Attrition was due mainly to changes in phone numbers/addresses or lack of time and amounted to *n* = 9 for the adoptive group and *n* = 18 for the community group. There were no differences between retained and nonretained participants. In two cases, only the parents participated, but not the adolescents.

An additional cross‐sectional sample of adolescents adopted from Russia (*n* = 38) of the same age range was included in this study. Families in a regional administrative record of internationally adopted families were sent letters with information about the study and invitations to enroll, along with the investigators' contact details.

The community families were recruited from 10 schools of varied socioeconomic backgrounds in the same city where most of the adoptive families from the longitudinal sample lived. Both the performance measures and parent reports were collected during two‐hour home visits by one or two trained psychologists, in which parents and adolescents were informed about the study and provided informed consent before responding to the study measures. The study was approved by the regional Ethics in Biomedical Research Andalusian Committee following international regulations on biomedical research with human subjects (07/18/2016, ID: 0147‐N‐16). The first language for all participants was Spanish, and all the measures and study procedures were administered in this language.

### Measures

#### Data collection sheet

Adoptive parents provided information through a data collection sheet on the presence of different disorders and conditions in the adopted adolescents (e.g., ADHD, medical problems, developmental delay… see Table S2 in the Supplementary Material for a full list). A question on FASD diagnosis or prenatal alcohol exposure was included as well: “Does your child have any of the following conditions? Fetal alcohol syndrome/fetal alcohol exposure,” with “Yes/No” response options. Those adolescents whose parents responded affirmatively to this question were classified as the parent‐reported FASD group. The direct assessment of sentinel facial features recommended in the main diagnostic systems (including the Canadian guidelines, Brown et al., 2019) was not part of our procedure. Our assessment included functioning in neurodevelopmental areas and parents' report of FASD and prenatal alcohol exposure. We consider the adequacy and limits of this approach in the Discussion section.

Adoptive parents also provided information on the number and types of services the adolescent was receiving at the time of assessment (e.g., speech therapist, psychotherapy, education specialist… see Table S1 in the supplementary material).

#### Social skills improvement system

The Social Skills Improvement System—Parent version (SSIS; Gresham & Elliott, 2008) is a widely used parent‐reported questionnaire assessing children's social skills and problem behaviors. The total social skill score assesses different types of social skills such as communication, cooperation, assertiveness, or responsibility (46 items; e.g., “Takes turns in conversations”), the internalizing problems score reflects depressive and anxious symptomatology (10 items; “Looks anxious in the presence other people”), the externalizing problems score assess common behavior problems such as aggressive or antisocial behaviors (12 items, e.g., “Is aggressive on people or objects”) and the autism spectrum score evaluates behaviors characteristic of autism spectrum disorder (15 items; e.g., “Makes repetitive movements”). The items are answered on a 4‐point Likert scale (0—*never* to 3—*almost always*). Cronbach's alpha was higher than 0.75 for all subscales.

#### Relationship problems questionnaire

The Relationship Problems Questionnaire (RPQ; (Minnis et al., 2002)) is a parent‐reported questionnaire assessing children's inhibited attachment disorder and disinhibited social engagement symptoms on a 4‐point Likert scale (0—*not at all like my child* to 3—*exactly like my child*). We used the disinhibited social engagement symptoms subscale comprising four items (e.g., “Gets too physically close to strangers”). Cronbach's alpha was 0.76.

#### Language20Q scale

Language difficulties were measured through the Language20Q scale (Ottem, 2009), a parent‐reported questionnaire including 20 items measuring difficulties in everyday language in children and adolescents, including expressive, receptive, and semantic language impairment (e.g., “Your child has trouble understanding the meaning of common words”). Items were answered on a 5‐point Likert scale (from 1—*not at all* to 5—*completely true*). Cronbach's alpha was 0.95.

#### K‐Bit

The Kaufman Brief Intelligence Test (K‐BIT; (Kaufman & Kaufman, 1990) is a widely used short performance test for measuring intelligence in children (≥4 years). It includes an abstract reasoning test (similar to Raven matrices) measuring fluid intelligence. In this study, research assistants administered this test directly to the adolescents. The measure provides standardized norms for age and gender and has well‐established reliability and validity.

#### CANTAB

The Cambridge Neuropsychological Testing Automated Battery (CANTAB; Luciana & Nelson, 2002) was used to measure adolescents' sensorimotor skills, attention, executive function, and working memory. The battery consists of a computerized series of tests on a touchscreen, visually attractive and entertaining for children and adolescents. The following tasks were administered to the adolescents by research staff in the same order (lasting typically around 40 min):

##### Motor Screening Task (MOT)

The MOT assesses sensorimotor deficits that would affect the collection of valid data in other tasks by requiring participants to touch as quickly as possible a flashing cross appearing at different locations on the screen. The score of visual‐motor latency (time to touch the cross) was used as an index of fine sensorimotor skills (lower scores show better performance).

##### Rapid visual information processing (RVP)

This is a test of visual sustained attention. In a white box, in the center of the screen, single digits appear at a rate of 100 digits/min. Participants must detect a series of target sequences (e.g., 3, 5, 7) and register responses by touching a blue button. Total misses (based on signal detection theory) were used as the outcome score for sustained attention (lower scores show better performance).

##### Stockings of Cambridge (SOC)

This test is a version of the Tower of London spatial task assessing planning, a complex executive function. Adolescents are shown two displays containing three colored balls held in stockings. The participant must use the balls in the lower display to match the pattern in the upper display, following certain rules and using the fewest number of moves possible. The outcome score used was the number of moves executed to complete problems that can be solved in five moves (lower scores show better performance).

##### Spatial working memory (SWM)

The SWM task assesses the ability to retain spatial information and use it in working memory. Several colorful squares are displayed on the screen, and the child is required to locate a blue token hidden in every box, following the rule that after a token has been found in a box, that box will not contain any tokens in the future. The number of boxes increases gradually, until a total of eight boxes. The outcome score was a total number of errors (touching boxes that were already found to be empty or contain a token). Lower scores show a better performance.

#### Assessment of impairment in neurodevelopmental domains

The Canadian Guidelines for Diagnosis for FASD consider impairment in three of the 10 following neurodevelopmental domains as one of the indicators of FASD diagnosis: motor skills, neuroanatomy/neurophysiology, cognition, language, academic achievement, memory, attention, executive function, affect regulation, and adaptive behavior/social skills/social communication (Cook et al., 2016). Our assessment evaluated 9 of these 10 domains (all except academic achievement).

The Canadian Guidelines consider evidence of impairment having a score of 2 *SD* below the standardized or community mean. Since we had a community control group, the mean and the *SD* from the community group were taken as a reference to calculate the cutoff scores for impairment in each area. See Table 2 for the measures used for each neurodevelopmental domain and Table S1 in the supplemental material for the cutoff scores used. The −2 *SD* cutoff was selected to indicate impairment, more stringent than other diagnostic guidelines using a −1.5 *SD* cutoff, because of the high‐risk nature of the internationally adopted sample (Hoyme et al., 2016).

**TABLE 2:** Impairments in neurodevelopmental areas in adopted adolescents with parent‐reported FASD, adopted adolescents without FASD, and community adolescents.

		Adopted with FASD (*n* = 14)	Adopted without FASD (*n* = 55)	Community (*n* = 30)	Chi‐square test^b^	
	Measure used	*n* (%)^a^	*n* (%)^a^	*n* (%)^a^	*Χ* ^2^ (1)	*p*
Deficits in neurodevelopmental areas						
Motor skills	CANTAB MOT	4 (28.6)	14 (25.5)	1 (3.3)	0.06	0.813
Neuroanatomy	Head circumference	7 (50.0)	16 (29.1)	1 (3.3)	2.20	0.138
Cognition	K‐Bit—Fluid intelligence	3 (21.4)	14 (25.5)	1 (3.3)	0.10	0.755
Language	Language 20Q	10 (71.4)	23 (41.81)	2 (6.7)	**3.92**	**0.048**
Attention	CANTAB RVP	1 (7.1)	12 (21.8)	1 (3.3)	1.57	0.210
Executive function	CANTAB SOC	4 (28.6)	17 (30.9)	2 (6.7)	0.029	0.865
Memory	CANTAB SWM	9 (64.3)	11 (20.0)	1 (3.3)	**10.63**	**0.001**
Affect regulation	SSIS—Internalizing problems	9 (64.3)	8 (14.5)	1 (3.3)	**14.87**	**<0.001**
Social skills	SSIS—Social skills	5 (35.7)	6 (10.9)	0 (0.0)	**5.12**	**0.024**
Deficits in three areas or more		11 (78.6)	20 (36.4)	0 (0.0)	**8.035**	**0.005**

Abbreviations: CANTAB, Cambridge automated neuropsychological testing automated battery; FASD, fetal alcohol spectrum disorder; MOT, Motor Screening Test; RVP, rapid visual information processing; SOC, Stockings of Cambridge; SSIS, Social Skills Improvement System; SWM, spatial working memory. Bolded values indicates statistically significant differences between the adoptes FASD and non‐FASD groups in the chi square tests.

^a^ Column percentage.

^b^ Chi‐square tests compare only the FASD and the non‐FASD‐adopted groups.

#### Physical development

The adolescent's orbitofrontal head circumference was measured with a tape measure by the researchers, who were trained to conduct this measurement. Adolescents reported their height and weight as measured using calibrated professional anthropometric measurement devices in local pharmacies. Standardized scores for age and gender based on Spanish multicentric population norms were obtained. These standards differ only slightly from the broadly used WHO growth tables (Guerrero‐Fernandez, 2020; Sánchez González et al., 2011).

### Data analyses plan

Missing data ranged from one to four cases (1.0%–4.0%) in all study variables except for the Language 20Q scale, in which it ranged from eight (8.1%) to 12 cases (12.1%). Missing data were considered missing completely at random (MCAR) following Little's MCAR test (*Χ* ^2^ = 286.099, df = 263, *p* = 0.157; See Table S1 for missing data details). In all cases except for the Language 20Q scale, there was unit‐level missingness (the whole measure was missing). Considering the missingness unit‐level nature, the small sample size, the small percentage of missingness, and the MCAR assumption, we decided to use listwise deletion using the available *n* for each analysis, which should not introduce bias in these circumstances (Dong & Peng, 2013). In the case of the Language 20Q scale, there was unit‐level missingness in eight cases and item‐level missingness for four items (for one or two additional cases). We used the expectation maximization (EM) algorithm to impute item‐level missing values in this scale, using all the scale items.

Chi‐squared tests were used to compare the adopted adolescents with and without FASD on the presence of impairment in neurodevelopmental domains, comorbid conditions, and service use. To compare the two adopted subgroups and the community group on the continuous variables of psychosocial, neurocognitive, and physical development, we conducted a one‐way ANOVA per variable and Games–Howell post hoc tests (not assuming equal variance). Given that the assumption of normality was not met for most variables, nonparametric bootstrapped bias‐corrected accelerated confidence intervals based on 2000 samples were performed as measures of statistical robustness. This technique has been shown to perform well with heteroscedastic and nonnormal data (Carpenter & Bithell, 2000). Cohen's *d* was calculated as a measure of effect size for the post hoc comparisons when adequate.

## RESULTS

### Preliminary analyses

We compared the adopted subgroups and the community group on several potentially relevant covariates, including age at assessment, age at adoption (for the adopted subgroups), gender, caregiver educational level, and family structure using one‐way ANOVA for continuous variables (age and age at adoption) and chi‐squared for categorical variables (gender, caregiver's educational level and family structure). Only the caregiver's educational level differed between groups, reaching statistical significance, with both adopted groups showing a higher proportion of caregivers with higher education levels (see Tables S3 and S4 in the Supplementary material for full details). Below, we explain our approach for statistical control of this variable in the main analyses.

### FASD and neurodevelopmental impairment in eastern European internationally adopted adolescents

Fourteen (20.3%) of the adopted adolescents were identified by their parents as having FASD or prenatal alcohol exposure. As mentioned above, adopted adolescents with or without parent‐reported FASD did not differ in age, gender, or age at adoption. Table 2 presents the percentage of adolescents with impairment in neurodevelopmental areas and the proportion of adolescents showing impairment in three or more areas in each subgroup. Neurodevelopmental impairments were pervasive in the group with FASD, who presented a higher proportion of difficulties than adopted adolescents without FASD in language, working memory, affect regulation, and social skills. While these problems were also frequent in the adopted group without FASD, to a lesser degree, the impairments were infrequent in the community comparison group. Impairment in neuroanatomy as indicated by having a head circumference 2 *SD* below the community group mean was present in 50% of the adolescents in the FASD group but also in 29.1% of the adopted adolescents without FASD.

In total, 31 adopted adolescents presented impairment in three neurodevelopmental areas or more (44.9% of the adopted adolescents). Eleven of the 14 (78.6%) adopted adolescents with FASD presented impairments in three areas or more, a higher proportion than the 20 of the 55 (36.4%) in the adopted non‐FASD subgroup. None of the community adolescents presented three impairments or more.

#### Comorbidity and service use

We tested the differences in the presence of several comorbid health and developmental problems among adopted adolescents with and without FASD. Those with FASD presented a higher proportion of developmental delay (*χ* ^*2*^ (1) = 7.93, *p* = 0.005), behavior disorder (five of the seven adolescents with this condition had FASD; *χ* ^*2*^ (1) = 12.60, *p* ≤ 0.001), and learning disability (*χ* ^*2*^ (1) = 8.04, *p* = 0.005). The subgroups did not differ in the prevalence of speech disorders or medical problems. Nine of the 14 adolescents (64.3%) with FASD had been diagnosed with ADHD, but the proportion was also high in the adopted adolescents without FASD (21/55, 38.2%; *χ* ^*2*^ (1) = 3.09, *p* = 0.079). Adopted adolescents in the FASD subgroup had an average of more than two (*M* = 2.57, SD = 1.65) comorbid disorders, whereas adopted adolescents without FASD had an average of around one (*M* = 1.15, SD = 1.15). This mean difference was statistically significant (*t*(16.32) = −3.05, *p* = 0.007, *d* = 1.00).

Regarding the use of mental health, education, or other services, adopted adolescents in the FASD subgroup received more services than those without FASD, including counselors (*χ* ^*2*^ (1) = 11.15, *p* ≤ 0.001), psychologists (*χ* ^*2*^ (1) = 6.09, *p* = 0.014), psychiatrists (*χ* ^*2*^ (1) = 4.44, *p* = 0.035), educational specialists (*χ* ^*2*^ (1) = 5.92, *p* = 0.015), or psychotropic medication for emotional and behavioral problems (*χ* ^*2*^ (1) = 4.99, *p* = 0.026).

### Psychosocial, neurocognitive, and physical profile of adopted adolescents with and without FASD in comparison with community adolescents

We compared the adopted adolescents with and without reported FASD and the community adolescents in their psychosocial adjustment, neurocognitive, and physical development using one‐way ANOVAs and Games–Howell post hoc tests. Given that the three groups differed in the proportion of caregivers with higher education, following the backdoor path criteria, it was checked whether this variable was associated with all the tested psychosocial adjustment, neurocognitive, and physical development outcomes (i.e., whether participants differed in outcomes based on this variable). The participants differed only in weight based on the caregiver's educational level, so we considered this covariate in the analysis with weight as the dependent variable.

Regarding psychosocial adjustment (Table 3), the adopted adolescents with FASD had a higher degree of difficulties than the adopted adolescents without FASD and the community group in most variables, as indicated by particularly large effect sizes for internalizing problems, autism spectrum behaviors, and externalizing problems. Adopted adolescents with FASD also had worse social skills than the community adolescents (large effect size) and the rest of the adopted adolescents (medium effect size). Disinhibited social engagement seemed to be less related to FASD, since it did not show statistically significant differences between groups in the ANOVA analysis.

**TABLE 3:** Between‐group comparison of psychosocial adjustment in adopted adolescents with parent‐reported FASD, adopted adolescents without FASD, and community adolescents.

	Adopted with FASD	Adopted without FASD	Community	*F* (2, 95)	Mean difference	95% Bca bootstrapped CI		Cohen *d*
	*M* (SD)	*M* (SD)	*M* (SD)			Lower bound	Upper bound	
Social skills	84.15 (21.62)	99.38 (17.74)	108.57 (14.67)	8.46***				
C–ANF					**8.85**	**1.30**	**17.00**	**0.55**
C–AF					25.48**	**11.86**	**39.32**	**1.44**
ANF–AF					**16.63**	**3.07**	**29.53**	**0.82**
Internalizing problems	10.85 (3.87)	5.29 (4.40)	4.13 (2.70)	14.14***				
C–ANF					−1.05	−2.79	0.57	−0.30
C–AF					−6.89***	**−9.15**	**−4.69**	**−2.17**
ANF–AF					−5.85***	**−8.19**	**−3.53**	**−1.29**
Autism spectrum behaviors	16.00 (5.02)	9.36 (5.50)	6.47 (4.17)	16.97***				
C–ANF					−3.48*	**−5.81**	**−1.11**	**−0.58**
C–AF					−10.46***	**−13.72**	**−6.49**	**−2.15**
ANF–AF					−6.98***	**−10.25**	**−3.89**	**−1.23**
Externalizing problems	11.85 (5.35)	7.80 (4.98)	5.97 (4.22)	5.66**				
C–ANF					−1.51	−3.96	0.920	−0.39
C–AF					−5.85*	**−9.34**	**−2.59**	**−1.28**
ANF–AF					−4.34*	**−7.49**	**−1.34**	**−0.80**
Disinhibited social behavior	2.64 (2.76)	1.37 (2.14)	0.87 (1.74)	2.31				

*Note*: Bolded values indicate mean differences which CIs do not include zero. Games–Howell post hoc analyses not assuming equality of variances were used.

Abbreviations: AF, adopted group with FASD; ANF, adopted group without FASD; Bca, bias corrected accelerated; C, community group; FASD, fetal alcohol spectrum disorder.

***p < 0.001, **p < 0.010, *p < 0.05.

Differences in neurocognitive development (Table 4) were less clear‐cut. Although there were differences between the community and the two groups of adopted adolescents, there were no differences in visual‐motor skills, SOC mean moves (an index of planning, a complex EF), and fluid intelligence between the adopted adolescents with and without FASD. The adopted adolescents with FASD displayed more difficulties in spatial working memory and language than the adopted adolescents without FASD.

**TABLE 4:** Between‐group comparison of neurocognitive development in adopted adolescents with parent‐reported FASD, adopted adolescents without FASD, and community adolescents.

	Adopted with FASD		Adopted without FASD		Community		*F* (2, 95)	Mean diff.	95% Bca bootstrapped CI		Cohen*d*
	*M* (SD)	*n*	*M* (SD)	*n*	*M* (SD)	*n*			Lower bound	Upper bound	
Visual‐motor latency	774.16 (128.65)	14	746.44 (198.48)	53	639.93 (93.67)	29	4.88*				
C–ANF								−106.51**	**−174.40**	**−42.96**	**−0.63**
C–AF								−134.23**	**−214.03**	**−61.46**	**−1.27**
ANF–AF								−27.72	−112.97	58.71	−0.15
RVP total misses	2.21 (1.42)	14	2.47 (2.45)	53	1.41 (1.52)	29	2.43				
SOC mean moves	7.38 (1.91)	14	7.67 (1.79)	52	6.01 (1.19)	29	9.64***				
C–ANF								−1.66***	**−2.23**	**−1.08**	**−1.04**
C–AF								**−1.37**	**−2.63**	**−0.30**	**−0.94**
ANF–AF								0.30	−0.90	1.39	0.16
SWM total errors	17.00 (9.52)	14	10.67 (7.88)	51	5.76 (5.57)	29	10.87***				
C–ANF								−4.91**	**−7.77**	**−2.20**	**−0.69**
C–AF								−11.24**	**−16.83**	**−5.97**	**−1.59**
ANF–AF								**−6.33**	**−12.12**	**−0.68**	**−0.77**
Language use difficulties	48.93 (21.60)	14	34.02 (13.22)	54	22.78 (5.29)	23	16.26***				
C–ANF								−11.24***	**−15.52**	**−7.29**	**−0.98**
C–AF								−26.15**	**−39.55**	**−15.14**	**−1.89**
ANF–AF								−14.91	−27.92	−3.35	−0.98
Fluid intelligence	100.07 (10.45)	14	100.02 (17.04)	53	110.53 (8.30)	30	5.80**				
C–ANF								10.51**	**3.86**	**17.17**	**0.72**
C–AF								10.46**	**2.45**	**18.48**	**1.16**
ANF–AF								−0.53	−7.19	7.64	0.00

*Note*: Bolded values indicate mean differences which CIs do not include zero. Games–Howell post hoc analyses not assuming equality of variances were used.

Abbreviations: AF, adopted group with FASD; ANF, adopted group without FASD; Bca, bias corrected accelerated; C, community group; FASD, fetal alcohol spectrum disorder; RVP, rapid visual information processing; SOC, Stockings of Cambridge; SWM, spatial working memory.

***p < 0.001, **p < 0.010, *p < 0.05.

As for physical development (Table 5), head circumference presented substantial differences between groups: the subgroup of adolescents with FASD had a mean *z* score of −2.04, that is, two standard deviations below the mean. This low score led to a large effect size in comparison with the community adolescents and a medium one compared to non‐FASD adopted adolescents, who also had a lower mean head circumference than community adolescents. Neither height nor weight differed substantially between groups. Since the caregiver's educational level was a confounding variable for weight, we also conducted between‐group (ANCOVA) analyses for weight controlling for this variable. The results are presented in Table S6 in the Supplementary Material. Given that the statistical conclusion did not change, we presented the results of the ANOVA analyses for weight, without controlling for the covariate, in Table 5.

**TABLE 5:** Between‐group comparison of physical development in adopted adolescents with parent‐reported FASD, adopted adolescents without FASD, and community adolescents.

	Adopted with FASD	Adopted without FASD	Community	*F* (2, 95)	Mean difference	95% Bca bootstrapped CI		Cohen*d*
	*M* (SD)	*M* (SD)	*M* (SD)			Lower bound	Upper bound	
Head circumference *Z* score	−2.04 (1.29)	−1.26 (1.34)	−0.31 (1.03)	10.51***				
C–ANF					0.96**	**0.46**	**1.45**	**0.78**
C–AF					1.73**	**0.96**	**2.47**	**1.55**
ANF–AF					**0.77**	**0.02**	**1.54**	**0.58**
Height *Z* score	−0.64 (1.49)	−0.24 (1.12)	0.07 (0.92)	2.01				
Weight *Z* score	−0.76 (0.76)	−0.36 (1.05)	0.04 (1.23)	2.91				

*Note*: Bolded values indicate mean differences which CIs do not include zero. Games–Howell post hoc analyses not assuming equality of variances were used.

Abbreviations: AF, adopted group with FASD; ANF, adopted group without FASD; Bca, bias corrected accelerated; C, community; FASD, fetal alcohol spectrum disorder.

***p < 0.001, **p < 0.010.

**FIGURE 1:**
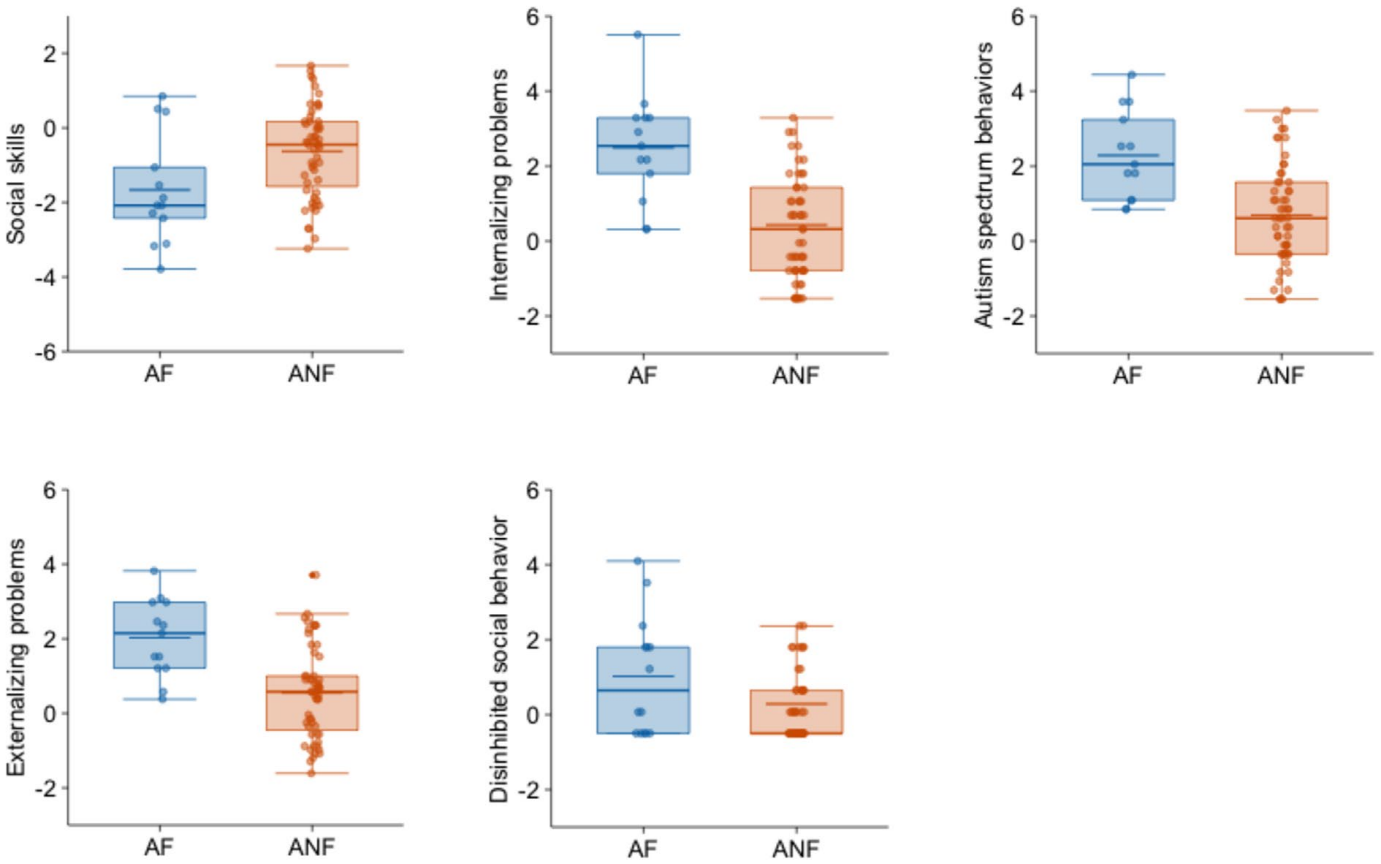
Standardized‐to‐norm means and distribution of psychosocial adjustment variables in adopted adolescents with and without FASD. Scores were standardized using the community group mean and SD. AF, adopted adolescents with FASD; ANF, adopted adolescents without FASD.

**FIGURE 2:**
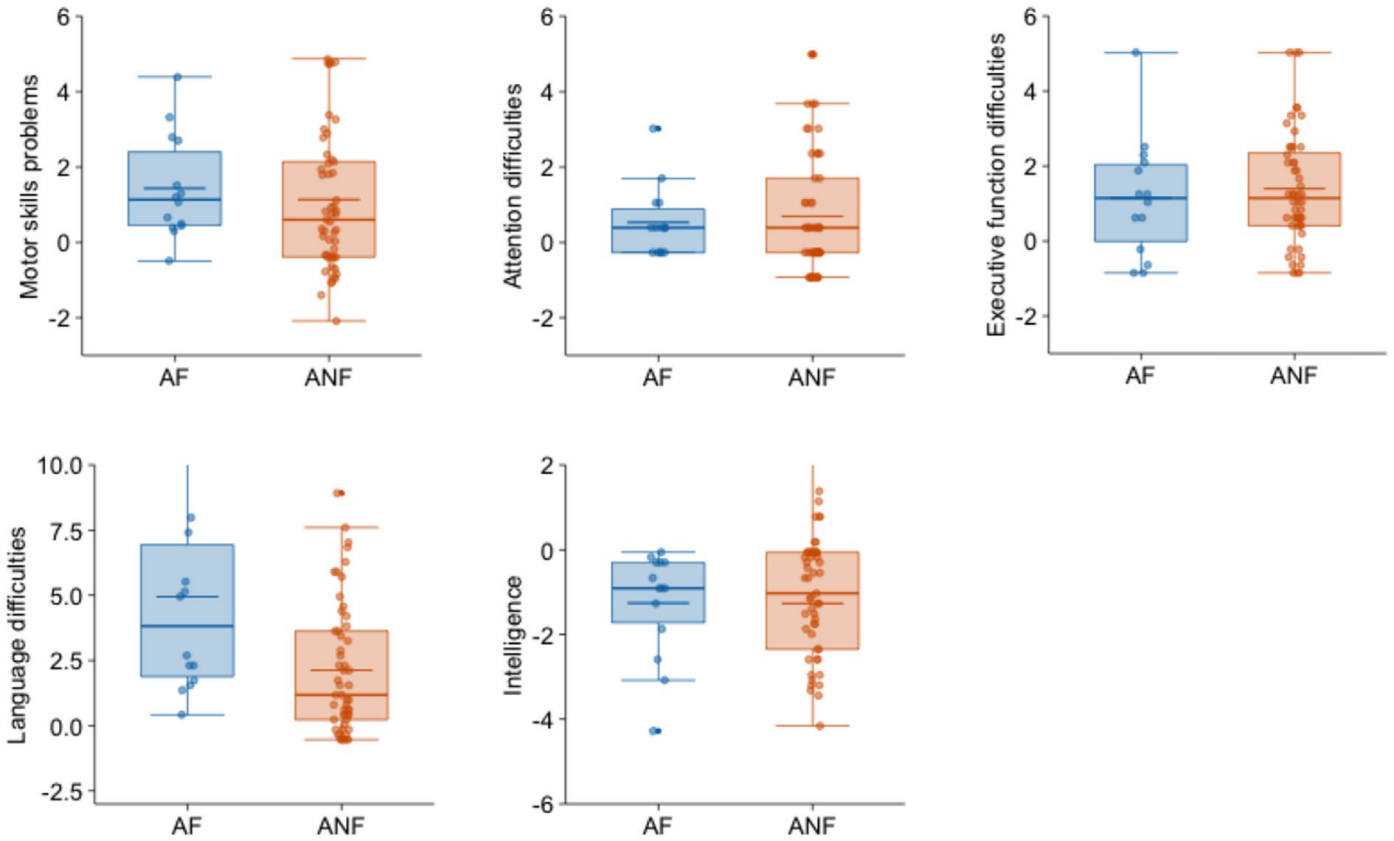
Standardized‐to‐norm means and distribution of neurocognitive variables in adopted adolescents with and without FASD. Scores were standardized using the community group mean and SD. AF, adopted adolescents with FASD; ANF, adopted adolescents without FASD.

**FIGURE 3:**
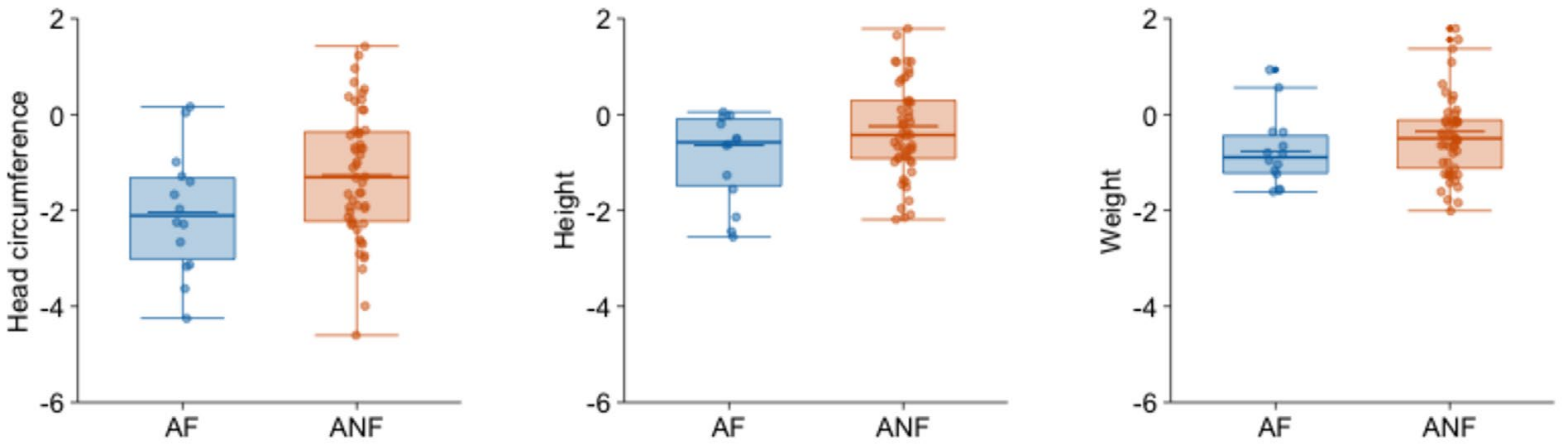
Standardized means and distribution of physical development variables in adopted adolescents with and without FASD. Standardized scores based on Spanish multicentric norms are presented (Sánchez González et al., 2011). AF, adopted adolescents with FASD; ANF, adopted adolescents without FASD.

Figure 1 shows a comparison of the adopted group with and without FASD in the psychosocial adjustment variables, while Figure 2 shows neurocognitive variables and Figure 3 physical growth variables. Figures 1 and 2 show the standardized‐to‐norm mean and distribution on each variable (scores were standardized using the community group mean and standard deviation). Scores in those figures, therefore, reflect how many standard deviations each adopted group differs from adolescents in the community group. Figure 3 uses standardized scores for head circumference, height, and weight based on Spanish multicentric norms (Sánchez González et al., 2011).

## DISCUSSION

In this study, a group of adopted adolescents of Eastern European origin with parent‐reported FASD or prenatal alcohol exposure was compared with adopted adolescents without documented or diagnosed FASD. Both groups were also compared with a group of community children in several dimensions, including neurodevelopmental impairments and psychosocial, neurocognitive, and physical development variables. Overall, adopted adolescents with FASD showed more complex profiles than adopted adolescents without FASD, including a higher proportion of impairments in neurodevelopmental domains, and more difficulties in psychosocial adjustment and neurocognitive development. Adopted adolescents without FASD also showed difficulties to a lesser degree, whereas severe problems (impairments in neurodevelopmental domains indicated by more than 2 SD from the mean) were rare in the community group.

The adoptive parents in our study responded to a question on whether their adopted adolescents presented FASD or had been exposed to alcohol prenatally, according to their children's records. Fourteen adolescents were reported to have FASD, 20.3% of the adoption subsample. This proportion is lower than that reported by previous studies assessing FASD incidence, including assessments of facial sentinel features in adopted children from Eastern Europe, which have reported the presence of FASD in around 50% of the adopted children assessed (Colom et al., 2021; Landgren et al., 2010). In their analysis of FASD among special subpopulations, Popova et al. (2019) reported a heterogeneous incidence in Russian orphanages, with the highest numbers in centers for children with developmental abnormalities. To the best of our knowledge, the participants in our study were not adopted from such orphanages.

Because our original research project focused on the catch‐up socioemotional and cognitive development of internationally adopted children and not on FASD specifically, we did not measure facial sentinel features and, therefore, were limited in our assessment of FASD following common diagnostic criteria (Brown et al., 2019; Cook et al., 2016). Specifically, any of the 20 adopted children without a FASD diagnosis who presented evidence of central nervous system impairment (impairment in ≥3 neurodevelopmental domains) could also present sentinel facial features and therefore be considered as having FASD according to the Canadian Guidelines diagnostic criteria (Cook et al., 2016), even in the absence of information about prenatal alcohol exposure. Consequently, it is possible that the inclusion of this key indicator would have increased the proportion of adopted children with FASD in our sample. Consistently with this argument, the two previous studies with Eastern European adopted individuals that included facial dysmorphology assessments along with neurodevelopment and history of prenatal alcohol exposure (Colom et al., 2021; Landgren et al., 2010) found a higher incidence of FASD (around 50%) than a previous study with adopted individuals from Poland to the Netherlands based on parent‐report of FASD diagnosis (31%; Knuiman et al., 2015).

The incidence of FASD in our study may also reflect factors like severity (only the most affected children have been those diagnosed) or access to practitioners with knowledge about FASD. Alternatively, some families with severely affected children may have declined to participate in the study. An additional argument is that our sample may present a lower proportion of children with special needs (including FASD) than later studies assessing internationally adopted children from Eastern Europe; over time, Russia (and other sending countries) increasingly favored international adoption of children with special needs (Popova et al., 2019; Selman, 2012).

Most of the children with reported FASD presented deficits in three or more neurodevelopmental domains, a requirement for an FASD diagnosis according to some guidelines (Cook et al., 2016). Deficits (indicated by scores more than two *SD* below the community group mean, or above for negative scores) were widespread in language, working memory, and affect regulation. These areas showed large differences between adopted adolescents with and without parent‐reported FASD, potentially indicating areas that are particularly affected by prenatal alcohol exposure and consistent with the pattern of neurobehavioral deficits found in individuals prenatally exposed to alcohol (Kingdon et al., 2016; Kully‐Martens et al., 2012; Mattson et al., 2019).

Adopted adolescents not diagnosed with FASD also presented deficits to a lesser degree, and 20 of them (36.4%) presented impairment in three or more neurodevelopmental domains, a common finding in adoption research (Brodzinsky & Palacios, 2023). Neurodevelopmental impairment was rare in the community group, and none of the community adolescents showed impairment in three areas or more. In combination, 31 of the 69 adopted adolescents (44.9% of the total adopted sample) presented impairments in three neurodevelopmental domains or more. Considering previous results on the incidence of FASD using multifaceted assessments among internationally adopted youth from Eastern Europe and the common underdiagnosis of FASD (Chasnoff et al., 2015; Colom et al., 2021), and given the lack of reliable information on prenatal alcohol exposure and other data such as facial dysmorphology and sentinel features, it is likely that at least some of the nondiagnosed cases in our study reflect unrecognized FASD. However, the presence of multiple developmental risk factors like early institutionalization in internationally adopted children and the nonspecificity of neurobehavioral difficulties associated with FASD mandates caution before assuming that every Eastern European internationally adopted individual with complex needs presents FASD. The alternative to underdiagnosis cannot be overdiagnosis, so interpretive prudence is of great importance.

As mentioned in the introduction, internationally adopted individuals have been exposed to an array of developmental risk factors and postnatal adversity, such as early institutionalization, poor nutrition, or disruptive caregiving relationships (Brodzinsky & Palacios, 2023; Johnson & Gunnar, 2011; Nelson et al., 2023; van IJzendoorn et al., 2020). These factors are known to have long‐term consequences in many of the areas that form the core difficulties of individuals with FASD, such as social relationships, self‐regulation and executive functions, or emotional and behavioral problems (Nelson et al., 2023; Rockhold et al., 2023). Therefore, it is certainly difficult, if not impossible, to disentangle whether the complex needs that may be present in some adopted individuals are because of prenatal alcohol exposure or because of other postnatal factors, something that other researchers studying this population have already pointed out (Colom et al., 2021; Knuiman et al., 2015; Landgren et al., 2010). It is equally true, however, that in some cases both risk factors were present, making the prognosis more negative.

In this study, the adopted adolescents with FASD presented more comorbid conditions such as learning disability, behavior disorder, or developmental delay than adopted adolescents without FASD, consistent with meta‐analytical findings (Popova et al., 2016). They also presented higher service use, including higher use of psychotropic medication, psychiatrists, or education specialists, reflecting the high need for services and support for individuals diagnosed with FASD (Bashista, 2022; Coons et al., 2018; Mohamed et al., 2020). In our comparison of the two adopted subgroups (with and without FASD) and the community group using a battery of psychosocial, neurocognitive, and physical measures, those with parent‐reported FASD had more severe internalizing and behavior problems, and difficulties in social communication, as indexed by large differences in autism spectrum behaviors and language use difficulties.

Regarding neurocognitive development, the pattern of between‐group differences was not as clear. Whereas in some areas, the adopted adolescents with reported FASD showed substantially worse outcomes than the adopted adolescents without FASD (e.g., language use problems or working memory), in other areas (e.g., intelligence, planning, or visual‐motor latency), the groups barely differed. In most of these areas, however, both adopted groups still showed lower scores than the community group, with predominantly large effect sizes. Regarding physical development, there were differences between groups only in head circumference, with both adopted groups showing much smaller mean head circumferences than the community group. The adopted adolescents with parent‐reported FASD showed an even smaller mean head circumference than those without FASD (−2.04 vs. −1.26), strongly suggestive of deficits in brain growth. Overall, the pattern of complex needs in mood or behavior regulation, certain neurocognitive areas, language, and social communication detected in adopted adolescents with parent‐reported FASD is largely consistent with the neurobehavioral profile of individuals diagnosed with this condition (Cook et al., 2016; Kully‐Martens et al., 2012; Mattson et al., 2019; Popova et al., 2023).

In summary, the two groups of Russian adoptees shared a preadoptive adversity that included institutionalization during early childhood, with neuropsychological and behavioral sequelae similar to those described in previous literature. Of these, the subgroup in which FASD was identified presented a profile of greater difficulties in social communication, working memory, language, and self‐regulation, as well as in psychosocial adjustment (including internalizing problems, autism spectrum behaviors, and externalizing problems) and in brain growth as manifested in a more compromised head circumference. With the necessary interpretative cautions derived in part from the limitations that we will now underline, the presence of FASD among adoptees calls for a more refined and complete diagnosis, as well as attention and support to the children and their families that are commensurate with their needs.

### Limitations and future directions for research

The major limitation of our study was the reliance on the information provided by adoptive parents on the FASD diagnosis or prenatal alcohol exposure of their adoptive children. Unfortunately, we were unable to obtain further information or confirmation on the degree of prenatal alcohol exposure, as it is so often common with samples of international adoptees. Furthermore, the assessment of facial dysmorphology associated with FASD was not part of our study procedure. We are aware of the limits of this approach and of the different incidence of FASD that a more thorough examination may have revealed (Chasnoff et al., 2015).

On the contrary, we believe the strength of our study lies in the in‐depth assessment of psychosocial, neurocognitive, and physical growth domains, providing a complete neurobehavioral profile based on standardized measures. We were able to include Eastern European internationally adopted adolescents with and without reported FASD and to compare both groups to community control adolescents who provided a reference point for typical development. Future research should assess the presence of FASD in Eastern European adoptees (as well as in those adopted elsewhere) using interdisciplinary approaches. However, even with a multidisciplinary assessment, it seems rather complicated to be certain of a FASD diagnosis with internationally adopted youth, considering that (a) the neurobehavioral difficulties associated with FASD are nonspecific (Lange et al., 2019), (b) neurobehavioral impairment associated with FASD is often present even in the absence of sentinel facial features (Popova et al., 2023; Wozniak et al., 2019), (c) information about preadoption experiences and obstetric records are usually not available and of questionable validity, and (d) internationally adopted youth have been exposed to other developmental risk conditions for neurobehavioral development (e.g., institutionalization, poor pre‐ and postnatal nutrition, etc.; van IJzendoorn et al., 2020).

Although our sample size was small for the subgroups comparison, the between‐group differences in variables of interest were so large and consistent that it seems clear that systematic and consistent differences were present, regardless of the potentially lower robustness of statistical analyses when comparing subgroups of a small (sub)sample size. An additional limitation is that we did not include assessments of adolescents' positive aspects and strengths. Research tends to focus on the deficits and difficulties of individuals with FASD, but it has also shown that individuals with FASD also present strengths such as persistence, self‐awareness, and social motivation, which may help them to overcome the significant adversities they have experienced (Flannigan et al., 2021; Kautz‐Turnbull et al., 2022).

## CONCLUSIONS AND IMPLICATIONS FOR PRACTICE

Widespread impairments in neurobehavioral domains such as memory, affect regulation, or language were present in Eastern European internationally adopted adolescents who were reported to have a FASD diagnosis. However, although to a lesser degree, impairments were also present in a substantial proportion of adopted adolescents not diagnosed with FASD. Regardless of the actual FASD status of those adopted adolescents with widespread impairments, almost half of the sample of Eastern European internationally adopted adolescents presented a complex profile, characterized by severe deficits in key neurocognitive and socioemotional areas. While the difficulties found in self‐regulation, social communication, and neurocognitive domains were strongly linked to a FASD diagnosis, we also recognize the nonspecificity of this neurobehavioral phenotype. The exposure of postinstitutionalized adopted youth to an array of developmental risk factors makes it difficult to be certain that these difficulties are caused by FASD or prenatal alcohol exposure.

Nevertheless, it is important to keep increasing the knowledge about FASD among practitioners who may work with adoptive families, and especially to provide adequate support and services for families, given the need for services and support for a child or an adolescent with FASD. As the best practices in this area recommend, services should be tailored to each person's limitations and strengths. There are a variety of services that can be helpful to individuals presenting FASD and their families, from positive behavior support to social skills training, executive functions interventions, respite care for caregivers, or targeted pharmacological interventions (Petrenko & Kautz‐Turnbull, 2021; Popova et al., 2023).

FASD is a lifelong condition that implies a complex neurobehavioral profile and a high need for services and support. However, there is much that can be done to identify and support children and youth with FASD and their families. Improving knowledge about this condition and its associated needs is an important step in that direction.

## CONFLICT OF INTEREST STATEMENT

The authors have no relevant financial or nonfinancial interests to disclose.

## Supporting information


Tables S1–S6

## Data Availability

The data supporting this study's findings are available from the corresponding author upon reasonable request.
