# Supplementary material for: Psychosocial, neurocognitive, and physical development in Eastern European adopted adolescents with and without fetal alcohol spectrum disorder
Source: Alcohol Clin Exp Res (Hoboken). 2025 Apr 27;49(6):1248–62. doi: 10.1111/acer.70068 (PMC12174492; doi:10.1111/acer.70068)
Supplement: Supplementary file 1 — Tables S1–S6 [file ACER-49-1248-s001.docx]

**Table S1**

*Psychosocial, Neurocognitive and Physical Development Areas Assessed, Measures Used and Cut-Off Scores for Neurodevelopmental Impairment*

| **Construct** | **Measure** | **Type of measure** | **Score used** | **Canadian Guidelines neurodevelopmental domain** | **Cut-off for impairment** |
| --- | --- | --- | --- | --- | --- |
| **Psychosocial adjustment** |  |  |  |  |  |
| Social skills | Social Skills Improvement System | Parent-report | Total social skills | Social skills | Below 76.70 |
| Internalizing problems |  |  | Internalizing problems subscale | Affect regulation | Above 9.71 |
| Autism spectrum behaviors |  |  | Autism spectrum subscale | - | - |
| Externalizing problems |  |  | Externalizing problems subscale | - | - |
| Disinhibited social behavior | Relationship Problems Questionnaire |  | Disinhibited social behavior subscale | - | - |
| **Neurocognitive development** |  |  |  |  |  |
|  | CANTAB battery | Performance tests |  |  |  |
| Fine motor skills | MOT |  | Visual-motor latency (seconds) | Motor skills | Above 827.28 |
| Sustained attention | RVP |  | Total misses | Attention | Above 4.46 |
| Planning | SOC |  | Number of moves for 5-moves problems | Executive function | Above 8.39 |
| Spatial working memory | SWM |  | Total number of errors | Memory | Above 16.89 |
| Language difficulties | Twenty Questions on Language Skills | Parent-report | Total language difficulties score | Language | Above 34.85 |
| Fluid intelligence | K-Bit - Fluid intelligence | Performance test | Fluid intelligence standardized score | Cognition | Below 91.76 |
| **Physical development** |  |  |  |  |  |
| Head circumference |  | Direct measurement | Standardized orbitofrontal head circumference | Neuroanatomy | Below -2.00 |
| Height |  | Report from measurement in calibrated anthropometric devices | Standardized height | **-** | **-** |
| Weight |  |  | Standardized weight | **-** | **-** |

*Note.* MOT = Motor Screening Task, RVP = Rapid visual information processing, SOC = Stockings of Cambridge, SWM = Spatial working memory.

**Table S2**

*Missing Data Percentages per Variable*

|  |  | Missing | |
| --- | --- | --- | --- |
|  | *N* | Count | Percent |
| Language 20Q scale |  |  |  |
| Item 1 | 91 | 8 | 8.1 |
| Item 2 | 91 | 8 | 8.1 |
| Item 3 | 91 | 8 | 8.1 |
| Item 4 | 91 | 8 | 8.1 |
| Item 5 | 91 | 8 | 8.1 |
| Item 6 | 91 | 8 | 8.1 |
| Item 7 | 91 | 8 | 8.1 |
| Item 8 | 91 | 8 | 8.1 |
| Item 9 | 91 | 8 | 8.1 |
| Item 10 | 91 | 8 | 8.1 |
| Item 11 | 90 | 9 | 9.1 |
| Item 12 | 90 | 9 | 9.1 |
| Item 13 | 91 | 8 | 8.1 |
| Item 14 | 91 | 8 | 8.1 |
| Item 15 | 91 | 8 | 8.1 |
| Item 16 | 90 | 9 | 9.1 |
| Item 17 | 89 | 10 | 10.1 |
| Item 18 | 90 | 9 | 9.1 |
| Item 19 | 91 | 8 | 8.1 |
| Item 20 | 91 | 8 | 8.1 |
| Language skills total score | 87 | 12 | 12.1 |
| Social skills | 98 | 1 | 1.0 |
| Internalizing problems | 98 | 1 | 1.0 |
| Autism spectrum behaviors | 98 | 1 | 1.0 |
| Externalizing problems | 98 | 1 | 1.0 |
| Disinhibited social behavior | 98 | 1 | 1.0 |
| Visual-motor latency | 96 | 3 | 3.0 |
| RVP total misses | 96 | 3 | 3.0 |
| SOC mean moves | 96 | 3 | 3.0 |
| SWM total errors | 95 | 4 | 4.0 |
| Fluid intelligence | 97 | 2 | 2.0 |
| Head circumference | 96 | 3 | 3.0 |
| Height | 97 | 2 | 2.0 |
| Weight | 97 | 2 | 2.0 |

*Note***.** Little's MCAR test: *Χ*^2^= 286.099, *df* = 263, *p* = .157

**Table S3**

|  | Adopted with FASD  (*n* = 14) | Adopted without FASD  (*n* = 55) | Community  (*n* = 30 |  |
| --- | --- | --- | --- | --- |
|  | *M* (*SD*) | *M* (*SD*) | *M* (*SD*) | *F* (2, 96) |
| Age (years) | 15.75 (1.41) | 15.85 (1.33) | 16.11 (1.12) | 8.46 |
|  |  |  |  |  |
|  |  |  |  | *t* (67) |
| Age at adoption | 37.04 (15.03) | 31.85 (14.90) | - | -1.160 |

*Preliminary Analyses Comparing Adopted Groups, and Community Adolescents in Continuous Covariates*

|  | Adopted with FASD  (*n* = 14) | Adopted without FASD  (*n* = 55) | Community  (n = 30) |  | Chi-square test | |
| --- | --- | --- | --- | --- | --- | --- |
|  | *n* (%) | *n* (%) | *n* (%) |  | *Χ*^2^ (2) | *p* |
| Gender (male) | 8 (57.1) | 36 (65.5%) | 13 (43.3) |  | 3.89 | .143 |
| **Cg. With higher education level** | **9 (64.3)** | **43 (77.8)** | **16 (53.3)** |  | **6.48** | **.039** |
| Single family structure | 5 (35.7) | 13 (22.2) | 4 (13.3) |  | 2.885 | .236 |

*Note.* FASD = Fetal alcohol spectrum disorder.

**Table S4**

*Preliminary Analyses Comparing Adopted Groups, and Community Adolescents in Categorical Covariates*

*Note.* FASD = Fetal alcohol spectrum disorder.

**Table S5**

*Co-Morbid Problems and Service Use in Adopted Adolescents with Parent-reported FASD and Adopted Adolescents without FASD*

|  | Adopted with FASD  (*n* = 14) | Adopted without FASD  (*n* = 55) |  | Chi-square test | |
| --- | --- | --- | --- | --- | --- |
|  | *n* (%) | *n* (%) |  | *Χ*^2^ (1) | *p* |
| **Co-morbid conditions** |  |  |  |  |  |
| Medical problems | 2 (14.28) | 8 (14.54) |  | 0.00 | .980 |
| Developmental delay | 6 (42.86) | 6 (10.90) |  | **7.93** | **.005** |
| ADHD | 9 (64.29) | 21 (38.18) |  | 3.09 | .079 |
| Speech disorder | 1 (7.14) | 5 (9.09) |  | 0.05 | .817 |
| Behavior disorder | 5 (38.46) | 2 (3.64) |  | **12.60** | **.000** |
| Learning disability | 11 (78.57) | 20 (36.36) |  | **8.04** | **.005** |
| **Services use** |  |  |  |  |  |
| Counselor | 7 (50.00) | 6 (10.90) |  | **11.15** | **.001** |
| Psychologist | 12 (85.71) | 27 (49.09) |  | **6.09** | **.014** |
| Psychiatrist | 7 (50.00) | 12 (21.81) |  | **4.44** | **.035** |
| Educational specialist | 8 (57.14) | 13 (23.63) |  | **5.92** | **.015** |
| Speech therapist | 6 (42.86) | 18 (32.72) |  | .51 | .477 |
| Use of psychotropic medication | 10 (71.43) | 21 (38.18) |  | **4.99** | **.026** |

*Note.* FASD = Fetal alcohol spectrum disorder; ADHD = Attention deficit hyperactivity disorder. Bolded values indicate statistically significant results.

**Table S6**

|  | Adopted with FASD  (*n* = 14) | Adopted without FASD  (*n* = 55) | | Community |  |  |  | 95 % CI | |  |
| --- | --- | --- | --- | --- | --- | --- | --- | --- | --- | --- |
|  | EM | EM | EM | | *F* (2, 93) | *p* | Mean difference | Lower bound | Upper bound | Cohen*d* |
| **Weight *Z* score** | -0.79 | -0.31 | -0.40 | | 2.41 | .096 |  |  |  |  |
| C− ANF |  |  |  | |  |  | 0.27 | -0.34 | 0.87 | 0.26 |
| C−AF |  |  |  | |  |  | 0.75 | -0.84 | 1.58 | 0.71 |
| ANF−AF |  |  |  | |  |  | 0.48 | -0.29 | 1.26 | 0.46 |

*Between-group Analysis of Weight Controlling for Caregiver Educational Level using One-Way ANCOVA*

*Note.* Bonferroni post-hoc analyses were used. The model includes the proportion of caregivers with higher education as a covariate. Estimated marginal means (EM) controlling for the covariate are presented. The *F* test the effect of group on weight while controlling for the covariate. FASD = Fetal alcohol spectrum disorder, C = Community, ANF = Adopted without FASD, AF = Adopted with FASD.
